# Rivaroxaban and Aspirin in Peripheral Vascular Disease: a Review of Implementation Strategies and Management of Common Clinical Scenarios

**DOI:** 10.1007/s11886-019-1198-5

**Published:** 2019-08-30

**Authors:** Graham R. McClure, Eric Kaplovitch, Sukrit Narula, Vinai C. Bhagirath, Sonia S. Anand

**Affiliations:** 1 Division of Vascular Surgery, McMaster University, Hamilton, Ontario Canada; 2 Department of Clinical Epidemiology and Biostatistics, McMaster University, Hamilton, Ontario Canada; 3 Department of Medicine, University of Toronto, Toronto, Ontario Canada; 4 Population Health Research Institute, 237 Barton St East, Hamilton, ON L8L 2X2 Canada; 5 Department of Medicine, McMaster University, Hamilton, Ontario Canada

**Keywords:** Peripheral artery disease, Antithrombotics, Rivaroxaban, Aspirin

## Abstract

**Purpose of Review:**

Peripheral artery disease (PAD) affects an estimated 200 million people worldwide and is associated with significant cardiovascular morbidity and mortality. Cardiovascular risk is further increased among individuals with polyvascular disease, where either cerebrovascular or coronary artery disease is present in addition to PAD. In this review, we present common clinical scenarios encountered when managing patients with PAD and provide an evidence-based approach to prescribing optimal antithrombotics in this population.

**Recent Findings:**

The COMPASS trial recently demonstrated that rivaroxaban 2.5 mg BID + ASA daily significantly reduces major adverse cardiac and limb events in patients with PAD. Despite these advances, morbidity following MALE events remains high.

**Summary:**

With widespread approval by federal health regulators, the COMPASS regimen should be strongly considered in PAD patients who do not have a high bleeding risk. Implementing the COMPASS regimen in patients with PAD, along with other vascular risk reduction strategies, will have a substantial impact on reducing atherothromboembolic risk in patients with established vascular disease.

## Introduction

Over 200 million individuals, including approximately 10 million in the United States alone, are affected by peripheral artery disease (PAD) [1]. Due to their widespread atherosclerosis, those with PAD are at significantly increased risk of major adverse cardiac events (MACE), major adverse limb events (MALE), and mortality [2]. Following an index MALE, this risk increases even further [3•]. Aggressive secondary prevention strategies are required to lower this risk. In addition to antihypertensives, lipid-lowering therapy, antihyperglycemics, lifestyle improvements, and smoking cessation, optimizing antithrombotic therapy is essential. Histologic assessments of above and below knee amputation specimens due to vascular disease have demonstrated that a large proportion of vascular occlusions are mediated by thrombotic occlusive disease even in the absence of major atherosclerotic lesions [4]. For this reason, antithrombotic therapy has become a mainstay of pharmacologic therapy in PAD.

The COMPASS trial was a recent large multicenter international randomized control trial designed to assess the efficacy of an antithrombotic regimen consisting of low-dose rivaroxaban with and without aspirin in stable coronary artery disease (CAD) and PAD [5]. In this review, we will provide an overview of where COMPASS fits within the existing antithrombotic literature in PAD, as well as practical considerations for implementation of COMPASS regimen therapy in everyday practice.

## State of the Literature Preceding COMPASS

Secondary prevention for stable PAD targets multiple pathways. It incorporates risk factor modification (i.e., lipid-lowering therapy, glycemic optimization, blood pressure control, a walking program, and smoking cessation) in conjunction with antithrombotic therapy [6]. Over the last 15 years, there has been a proliferation of high-quality evidence from RCTs of antithrombotic agents in PAD, with greater than 40,000 patients included since 2007 [7]. While single antiplatelet therapy is the standard of care in stable PAD [6, 8, 9], recent evidence for targeting additional pathways beyond platelet inhibition has changed the vascular practitioner’s armamentarium [7].

### Single Antiplatelet Therapy

Current American Heart Association (AHA) and American College of Cardiology (ACC), European Society of Cardiology (ESC), and Canadian Cardiovascular Society (CCS) guidelines provide strong recommendations (class IA) for use of single antiplatelet therapy (SAPT) in the form of either aspirin (75–325 mg daily) or clopidogrel (75 mg daily) in patients with symptomatic PAD [6, 8, 9]. These recommendations are based on multiple randomized trials [10, 11]. A meta-analysis of RCTs performed by the Antithrombotic Trialist’s Collaboration which included 5269 PAD patients treated with aspirin (alone or with dipyridamole) versus placebo demonstrated a 22% reduction in odds for cardiac events (relative risk (RR) 0.88; 95% CI 0.76–1.04) associated with antiplatelet therapy, although no difference was observed in stroke or mortality [10]. Subsequent trials have tested whether more potent antiplatelet agents are more effective. The CAPRIE trial evaluated whether clopidogrel was superior to aspirin. CAPRIE was a large international RCT including 19,185 patients with a history of stroke, myocardial infarction (MI), or PAD randomized to aspirin 325 mg daily, versus 75 mg clopidogrel daily, 6452 of whom had a history of PAD [12]. CAPRIE demonstrated an 8.7% (95% CI 0.3–16.5) relative risk reduction conferred by clopidogrel over aspirin for the composite of stroke, MI, and vascular death. This was driven largely by the PAD subgroup where a 23.8% relative risk reduction was observed (95% CI 8.9–36.2). Safety profiles appeared similar between the two regimens with no difference in intracranial or GI bleeds.

The EUCLID randomized trial evaluated whether ticagrelor was superior to clopidogrel in 13,885 patients with symptomatic PAD. Patients were randomized to receive either ticagrelor 90 mg twice daily or clopidogrel 75 mg once daily [13]. No difference in the primary composite of ischemic stroke, MI, and cardiovascular death (10.6% vs 10.8%; HR 1.02; 95% CI 0.92–1.13) was observed between the two groups, or for key secondary outcomes including limb revascularization and acute limb ischemia.

### Dual Antiplatelet Therapy

The role for DAPT in stable PAD is less established. The ACC/AHA guidelines provide weak recommendations (class IIb) for consideration of DAPT use in PAD patients [6]. This is based largely on subgroup analysis of the CHARISMA trial which compared clopidogrel and aspirin to aspirin alone in patients with vascular disease. The overall trial result was neutral. In the PAD patients alone (*n* = 3096), there was also no significant reduction in composite cardiovascular death, MI, or stroke (HR 0.85; 95% CI 0.66–1.08; *p* = 0.18) [14, 15]. Subsequent to these guidelines however, the TRA2°P-TIMI 50 and PEGASUS-TIMI 54 trials were published which provide additional information regarding enhanced platelet inhibition in PAD patients.

TRA2°P-TIMI 50 was a large randomized trial (*n* = 26,449) which assessed vorapaxar, a strong inhibitor of platelets which functions as a protease-activated receptor-1 antagonist, compared to placebo, in patients with stable atherosclerotic vascular disease. The overall trial results showed a significant reduction in MACE (9.3% vs 10.5%; hazard ratio (HR) 0.87; 95% CI 0.80–0.94; *p* < 0.001), but a significant excess in major bleeding (4.2% vs 2.5%; HR 1.66; 95% CI 1.43–1.93; *p* < 0.001). It bears consideration that a substantial proportion (94%) of patients were receiving DAPT [16]. A subgroup analysis of patients with PAD (*n* = 5845) showed that the addition of vorapaxar to standard therapy (57.8% SAPT, 39.6% DAPT) significantly reduced the rate of peripheral limb revascularization in stable PAD patients (18.4% vs 22.2%; HR 0.84; CI 0.73–0.97; *p* = 0.017) [17]. However, an increase in moderate or severe bleeding (Global Use of Strategies to Open Occluded Arteries—GUSTO—classification) was also observed (4.2 vs 2.5%; HR 1.66; 95% CI 1.43–1.93) [16, 17]. Although this drug was approved by the FDA, its use is very limited in PAD patients [16, 17]. The PEGASUS-TIMI 54 trial randomized patients to the combination of aspirin and either ticagrelor 60 mg BID or 90 mg BID versus aspirin alone in patients within 1–3 years following MI (*n* = 21,165). There was a significant reduction in MACE at both doses individually (60 mg: 7.77% vs 9.04%; HR 0.84; CI 0.74–0.95; *p* = 0.004, 90 mg: 7.85% vs 9.04%; HR 0.85; CI 0.75–0.96; *p* = 0.008) as well as reduction to MALE when both active treatment arms were combined (0.46% vs 0.71%; HR 0.65; CI 0.44–0.95; *p* = 0.026). In the overall trial, significant increases in TIMI major bleeding were observed at both treatment doses (90 mg: HR 2.69; CI 1.96–3.70; *p* < 0.001 and 60 mg: HR 2.32; CI 1.68–3.21; *p* < 0.001). A subgroup analysis of patients with known PAD from PEGASUS (*n* = 1143) demonstrated that the ticagrelor 60 mg BID and aspirin regimen reduced MACE (14.1% vs 19.3%; HR 0.69; CI 0.47–0.99; *p* = 0.045) versus aspirin alone in PAD patients, and trended towards reduction in MALE without reaching statistical significance. When considering these effect estimates for both MACE and MALE, it is important to consider that the PAD subgroup analysis is underpowered to assess these outcomes.

### Full-Dose Anticoagulation

The Warfarin Antiplatelet Vascular Evaluation (WAVE) trial randomized the combination of vitamin K antagonist at moderate intensity (i.e., INR 2–3) and SAPT versus SAPT alone in patients with PAD of the lower extremities, carotid arteries, or subclavian arteries. This was the only large randomized controlled trial prior to COMPASS specifically designed to assess the efficacy of anticoagulation in stable PAD [7, 18•]. The trial randomized 2161 patients and demonstrated no significant difference in MACE or MALE, but a threefold increased risk of life-threatening bleeding was observed with full-dose warfarin in addition to antiplatelet therapy (4.0% vs 1.2%; RR 3.41; 95% CI 1.84 to 6.35; *p* < 0.001). This trial highlighted the hazard of the long-term use of moderate-intensity warfarin together with antiplatelet therapy in PAD patients. Taking this result together with the DUTCH Boa trial, which randomized 2690 patients following infrainguinal bypass to high-intensity vitamin K antagonist (INR 3–4.5) versus aspirin and showed no benefit to graft occlusion, and excess in life-threatening bleeding [19], limits the use of warfarin in chronic stable PAD patients.

### Rationale for the COMPASS Regimen

Rivaroxaban is a selective direct factor Xa inhibitor which has significantly lower rates of bleeding than vitamin K antagonists in clinical settings such as atrial fibrillation and venous thromboembolic disease [20, 21]. In the setting of recent acute coronary syndrome, the ATLAS TIMI-51 trial in which low dose rivaroxaban together with antiplatelet therapy was evaluated (> 90% DAPT) was superior to placebo with regard to reduction in MACE (HR 0.76; 95% CI 0.66–0.86; *p* < 0.001); however, it was associated with a significant increase in rates of major bleeding (2.1% vs 0.6%, *p* < 0.001) limiting its uptake into standard practice [22]. In a small subgroup of patients on SAPT at baseline however, the addition of rivaroxaban continued to show benefit without a significant difference in bleeding between both groups. In light of these results, the COMPASS trial was designed to test the efficacy and safety of low-dose rivaroxaban with or without aspirin in comparison to aspirin alone in patients with CAD or PAD.

### COMPASS Population

The COMPASS trial was a large, international, multicenter RCT which enrolled 27,395 patients with stable atherosclerotic vascular disease (including CAD and PAD) between 2013 and 2016 [5]. Patients were randomized (1:1:1) to receive either oral rivaroxaban 2.5 mg twice daily plus aspirin 100 mg daily, oral rivaroxaban 5 mg twice daily alone, or 100 mg PO aspirin daily. The trial demonstrated reduction in MACE defined as composite myocardial death, stroke, or MI with rivaroxaban plus aspirin (4.1% vs 5.4%; HR 0.76; 95% CI 0.66 to 0.86; *p* < 0.001) but an increase in major bleeding events (3.1% vs 1.9%; HR 1.70; 95% CI 1.40–2.05; *p* < 0.001). However, no difference in intracranial or fatal bleeding was observed. The rivaroxaban alone arm did not significantly reduce rates of MACE in comparison to aspirin alone, but did increase rates of major bleeding (2.8% vs 1.9%; HR 1.51; 95% CI 1.25–1.84; *p* < 0.001) [5].

Included in COMPASS were 7470 patients with PAD from 558 centers [5]. The COMPASS PAD population was composed primarily of patients who had either symptomatic peripheral vascular disease (55%), or carotid stenosis with or without previous intervention (26%). The remainder of patients included were asymptomatic PAD patients with known CAD and ABI < 0.9 [24].

PAD patients were required to meet one of the following criteria for enrollment [23•]:

1. Ongoing claudication symptoms associated with either
  - Reduced ABI < 0.9
  - Radiological evidence of vessel stenosis > 50%
2. Previous revascularization of the lower limbs by either open or endovascular repair
3. Previous amputation of a limb or foot due to vascular insufficiency
4. Documented CAD and an ABI < 0.9
5. Radiologic evidence of carotid artery stenosis > 50% or prior carotid revascularization

Unlike the COMPASS CAD population, PAD patients were not restricted based on age or additional vascular risk factors. Exclusion criteria for the trial were contraindications to low-dose rivaroxaban, including elevated bleeding risk, stroke within 1 month or any prior hemorrhagic stroke, severe heart failure (NYHA III/IV or EF < 40%), and eGFR < 15 mL/min. Patients with indications for dual antiplatelet, other non-aspirin antiplatelet, or oral anticoagulation therapy were also excluded.

Among COMPASS PAD patients, vascular risk factors including hypertension (79%), diabetes mellitus (44%), and current or former smoking history (74%) were common. The proportion of patients with concomitant CAD was 66%. Use of vascular protective agents such as antiplatelet therapy prior to randomization (87%), lipid-lowering agents (83%), and ACE-I/ARB (70%) was higher than is observed in registries.

### COMPASS Results

**Fig. 1:**
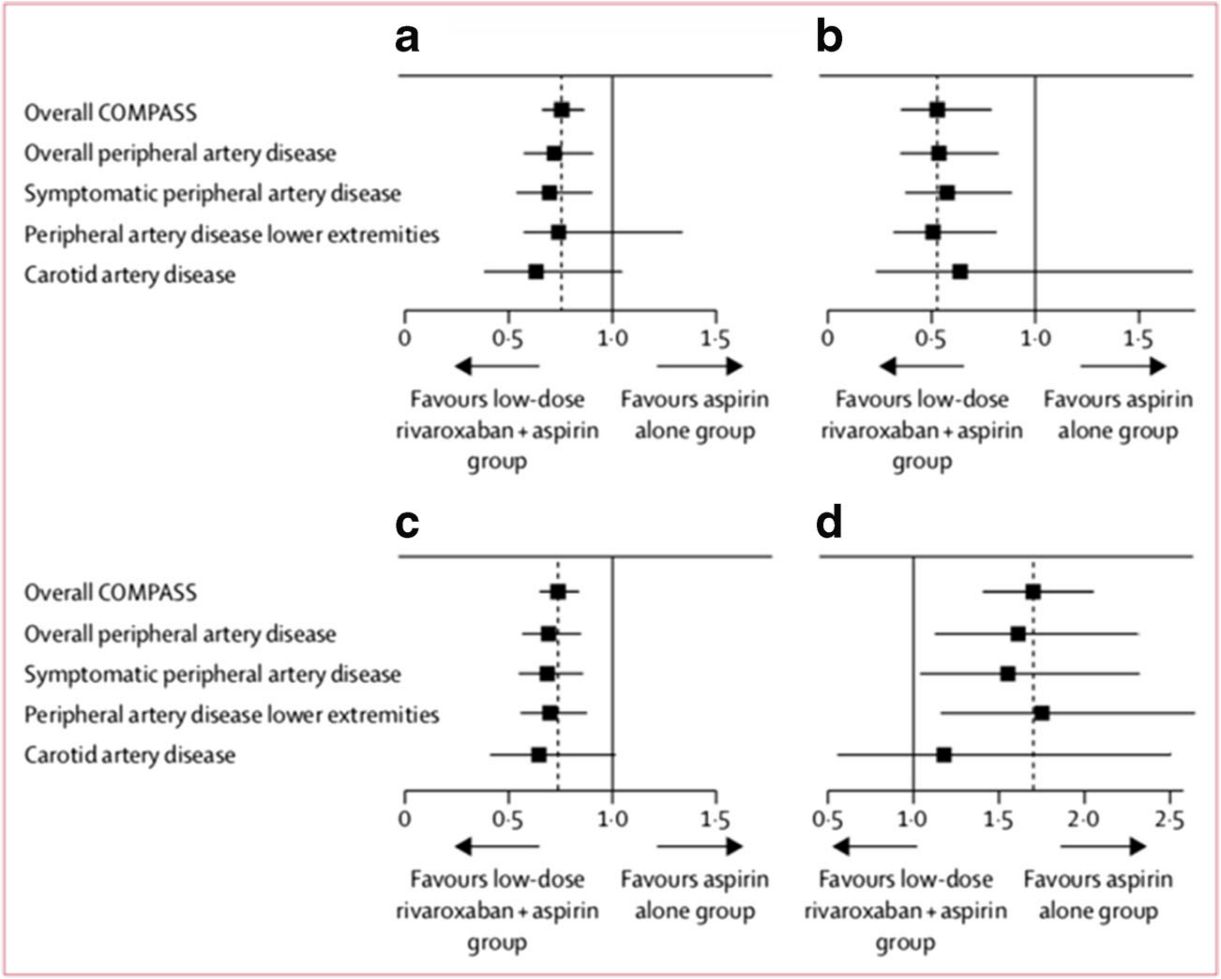
Analyses of primary and secondary outcomes—hazard ratios and 95% CI are shown for all subgroups of patients with peripheral artery disease for major adverse cardiac events (**a**) and major adverse limb events including major amputation (**b**), major adverse cardiac or limb events including major amputation (**c**) and for major bleeding (**d**). The dotted line indicates the point estimate for the overall COMPASS trial population (*n* = 27,395). (Reprinted from The Lancet: Anand SS, et al. Lancet Lond Engl. November 2017. doi:10.1016/S0140-6736(17)32409-1, with permission from Elsevier) [23•]

Patients with PAD who were randomized to receive rivaroxaban 2.5 mg twice daily plus 100 mg aspirin daily experienced significant reduction in MACE (cardiovascular death, MI, or stroke) (HR 0.72, 95% CI 0.57–0.90, *p* = 0.0047) and MALE (HR 0.54, 95% CI 0.35–0.82, *p* = 0.0037) when compared to those receiving aspirin alone [23•]. MALE in COMPASS was defined as any episode of severe limb ischemia leading to an intervention including acute limb ischemia, chronic limb ischemia, or major vascular amputation. Rates of acute limb ischemia alone were similarly reduced (HR 0.56; 95% CI 0.32–0.99; *p* = 0.042). While this regimen was associated with an increase in major bleeding events (HR 1.61, 95% CI 1.12–2.31, *p* = 0.0089) primarily from gastrointestinal sites, however there was no increase in critical organ bleeding, non-fatal intracranial bleeding, or fatal bleeding (HR 1.10, 95% CI 0.59–2.05). The net clinical benefit analysis, incorporating MACE, MALE, and severe bleeding events, maintained significant benefit in favor of the rivaroxaban plus aspirin arm (HR 0.72, 95% CI 0.59–0.87, *p* = 0.0008) [23•]. This benefit was consistent across patients from all included PAD subtypes (Fig. 1) [23•].

MALE was found to be an exceptionally poor prognostic factor. In the year following a MALE event, the risk of recurrent hospitalization (HR 7.21; *p* < 0.0001), amputation (HR 197.5; *p* < 0.0001), and death (HR 3.23; *p* < 0.001) all substantially increased [3•]. Patients who were randomized to rivaroxaban and aspirin also had a better prognosis after MALE than did patients randomized to aspirin alone [3•], suggesting that the type of MALE among patients on rivaroxaban and aspirin was of a lesser severity, i.e., non-occlusive thrombus compared to those on aspirin alone. Furthermore, this analysis demonstrated that in patients experiencing MALE, management of subsequent antithrombotic therapy was extremely heterogeneous, reflecting the clinical uncertainty surrounding the management of this condition. Post-MALE almost two-thirds of patients were maintained on their blinded study drug, while one-third of patients were removed from study drug and changed, in nearly equal proportions, to either SAPT, DAPT, or no therapy [3•].

**Fig. 2:**
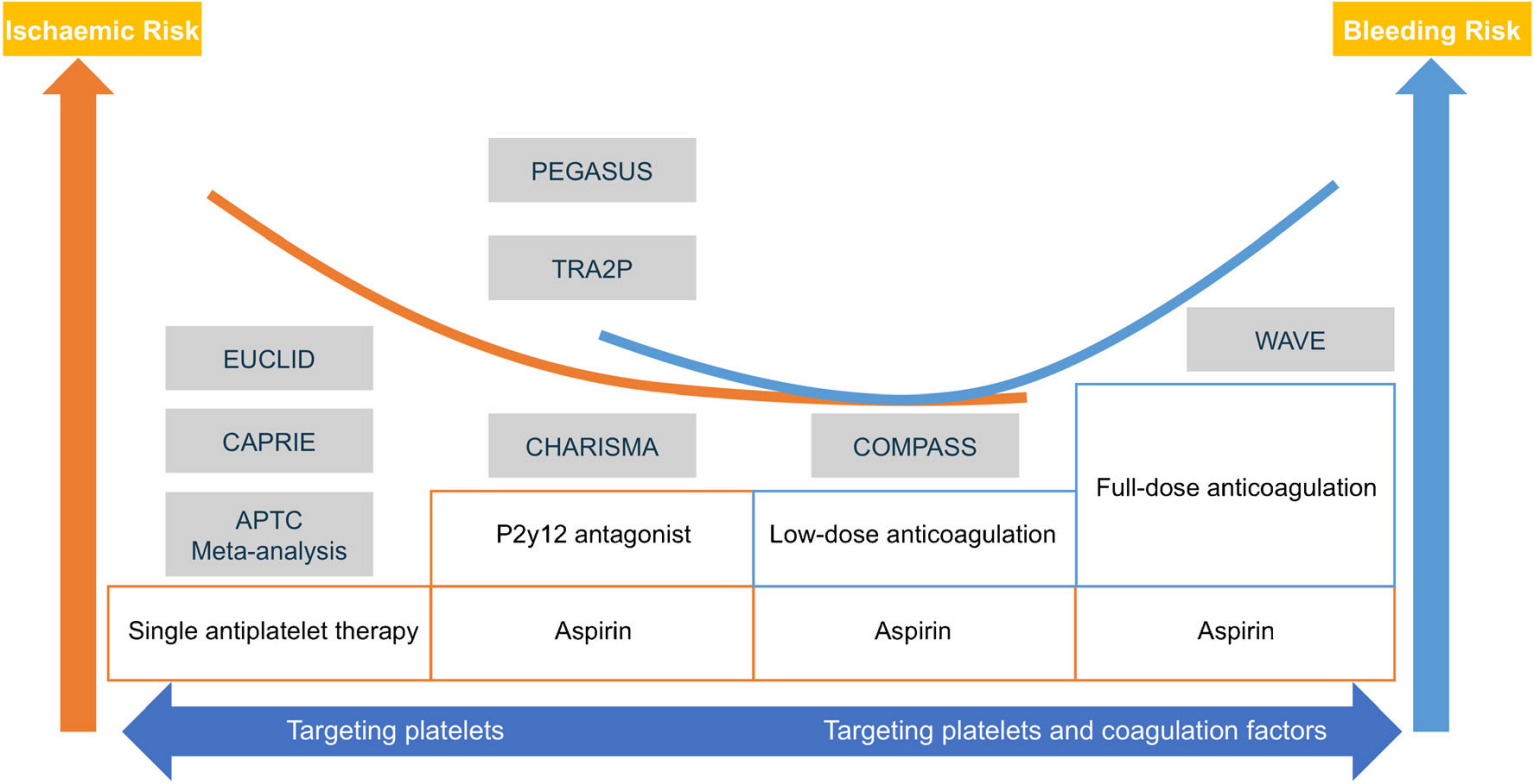
Visual summary of relative bleeding and ischemic risks associated with antithrombotic therapy regimes based on current available evidence

Of all trials assessing antithrombotic management in PAD, aspirin and rivaroxaban 2.5 mg BID is the only regimen, when compared to aspirin alone, which demonstrates reductions in both MACE and MALE in the setting of stable PAD, while also maintaining an acceptable safety profile (Fig. 2).

## Implementing COMPASS in Patients with PAD

### Who to Consider for Therapy

Dual pathway therapy with aspirin and rivaroxaban 2.5 mg BID is widely applicable to clinical practice. As described in the REACH registry, 68.4% of real-world PAD patients would meet eligibility criteria for COMPASS therapy [24]. In the REACH analysis, the most common reason PAD patients would be excluded from consideration to start low-dose rivaroxaban and aspirin therapy was high bleeding risk (51.8%), followed by the need for full-dose anticoagulation (44.8%) and the need for DAPT in the context of acute coronary syndrome (ACS) or percutaneous coronary intervention (PCI) (25.9%) [24]. COMPASS patients were seen to have lower absolute event rates than those in REACH, suggesting that the true effect estimate of ASA and low-dose rivaroxaban may be even greater in non-trial settings.

In patients eligible for COMPASS therapy, those at the highest risk of MACE and MALE should be the first considered for treatment. PAD patients with polyvascular disease, a history of heart failure, diabetes, or renal insufficiency are particularly susceptible to major vascular events and have greater absolute risk reduction in adding rivaroxaban 2.5 mg BID to aspirin as compared to aspirin alone [25•]. For example in PAD patients with concomitant CAD (i.e., 2 or more vascular beds affected vascular bed), the absolute risk reduction for vascular events is a striking 6%, compared to the 1.36% absolute risk reduction in patients with PAD alone [25•]. This number needed to treat is comparable to utilizing warfarin rather than aspirin in patients with non-valvular atrial fibrillation [26]. Clinical history to determine if your PAD patient has polyvascular disease, diabetes, or heart failure, in conjunction with basic bloodwork to screen for low eGFR, can help guide antithrombotic decision-making. While there is considerable residual risk reduction for COMPASS patients without high-risk features, an individualized discussion risk of MACE, MALE, and bleeding, contextualized within a patient’s access to medication, guides therapy.

### Monitoring After Initiation

As with any medication, clinical and laboratory monitoring are essential to avoid adverse events. Clinical and biochemical vigilance for bleeding while on the COMPASS regimen is prudent, with a particular focus on gastrointestinal and cutaneous bleeding, the areas where the COMPASS regimen has been shown to confer increased bleeding risk [5]. The COMPASS regimen has not been shown to increase intracranial or life-threatening hemorrhage [5]. Renal function should be monitored at least once per year, with consideration for more frequent measurement at times of acute illness or in patients with low baseline creatinine clearance [27]. In the setting of significantly reduced renal function (estimated glomerular filtration rate less than 15 mL/min), this regimen should be discontinued until renal function recovers. Additionally, rivaroxaban has the potential for drug interactions given its metabolism via cytochrome P450 3A4 and P-glycoprotein [28]. While only those with major interaction will be ineligible for COMPASS therapy, attentiveness towards interactions is needed with any newly prescribed therapy, particularly with antiepileptics and medications for HIV [28].

### Barriers to Implementation

The COMPASS regimen has been approved by the United States Food and Drug Administration, Health Canada, and the European Medicines Agency, among other medication regulatory bodies. Yet once a clinician determines that COMPASS therapy is appropriately indicated, barriers to prescriptions remain. Polypharmacy can limit patient adherence. Similar to other manifestations of atherosclerosis, an increasing number of medications are indicated to decrease morbidity and/or mortality in PAD. Statins and renin-angiotensin-aldosterone-receptor blockers are core therapies for vascular protection, though other antihypertensives, lipid-lowering agents, antihyperglycemics, and smoking cessation adjuncts are common as well given the pattern of comorbidity in vascular patients [6]. Regular exercise and smoking reduction should always be emphasized, and superfluous medications should be terminated in order to emphasize the use of efficacious vascular protective medications. PAD patients are undertreated and have approximately half the rate of guideline-based medication use as compared to their counterparts in CAD [29], despite the fact that optimal medical therapy for PAD patients has been shown to decrease MACE, MALE, and overall mortality [30, 31]. Antithrombotic therapy is one key aspect of medical management, but other risk factors should be modified including use of LDL, blood pressure, and glucose-lowering agents, as part of a global vascular risk reduction strategy [9].

The cost of rivaroxaban may represent a barrier to treatment for some PAD patients who do not have health insurance. However, cost-effectiveness analyses done using the COMPASS trial data indicate this therapy is cost effective for PAD patients due to avoided acute limb events, major amputations, and hospitalizations associated with these outcomes [32]. Once the drug becomes generic, its use will likely become more widespread. It is promising that an increasing number of private and public insurance agencies are providing coverage for low-dose rivaroxaban and aspirin as the importance of preventing costly cardiac and limb events becomes evident.

### Remaining Uncertainties

Some questions remain regarding the use of COMPASS therapy for patients with PAD. Foremost among them is the choice and intensity of antithrombotic therapy following major adverse limb events as this was not the primary question addressed in the COMPASS trial. Despite a 20.5% risk of amputation, 57.6% risk of cardiovascular hospitalization, and 8.3% risk of death in the year following a MALE, there was substantial heterogeneity in clinical management [3•]. The VOYAGER-PAD trial is underway and has randomized 6500 patients with symptomatic PAD undergoing infrainguinal endovascular and/or open revascularization to rivaroxaban 2.5 mg BID or placebo, on a background of aspirin (with allowances for a limited course of thienopyridine as well) [33]. The primary outcome includes MACE and MALE. While VOYAGER-PAD will provide valuable information regarding antithrombotic therapy following vascular intervention, further studies are required to clarify antithrombotic treatment following acute limb ischemia.

## Common Clinical Scenarios

Given the high risk of recurrent events in the polyvascular disease population, scenarios in which breakthrough thrombosis occurs or a separate indication for antithrombotic therapy arises will be common. We consider several of these scenarios below.

### Acute Coronary Syndrome or Percutaneous Coronary Intervention

In COMPASS, approximately 1.1% of patients in the rivaroxaban combination arm experienced acute coronary syndrome (ACS) per year [23•]. After ACS, current guidelines suggest use of dual antiplatelet therapy with low-dose aspirin and a P2Y12 inhibitor for at least 6–12 months [34]. When choosing a P2Y12 inhibitor, the PLATO trial supports use of ticagrelor over clopidogrel for the first 12 months after ACS [35]. This is associated with decreased overall mortality and MACE but an increase in non-procedure-related bleeding [35]. In light of this, it is reasonable to treat patients with PAD who experience ACS and are not at high risk of bleeding with ticagrelor and aspirin for 6–12 months. Beyond this 6–12-month period, a return to low-dose rivaroxaban plus aspirin should be considered. Patients with a history of ACS in COMPASS experienced a similar benefit with the combination regimen compared to aspirin as the overall group, including a decrease in overall mortality. This was consistent in patients who were enrolled < 2 years after MI [36]. By comparison, the PEGASUS trial demonstrated that 1–3 years post MI ticagrelor 60 mg twice daily in addition to aspirin reduced the risk of MACE, but not the risk of overall mortality compared to aspirin monotherapy [37]. It also increased risk of major bleeding events [37]. A small substudy of patients with both PAD and CAD however demonstrated a decrease in MACE and overall mortality, with no observed increase in major bleeding with the 60 mg dose alone [38]. Given this evidence, after a period of 6–12 months of dual antiplatelet therapy, either continued dual antiplatelet therapy with ticagrelor and aspirin or the COMPASS combination regimen can be employed. With increasing time since the ACS event, the COMPASS evidence becomes more compelling given the effect on mortality.

### Atrial Fibrillation

Patients with an indication for anticoagulation were excluded from the COMPASS trial. Given that RCTs have consistently shown reduced efficacy for stroke prevention in atrial fibrillation with lower compared to higher doses of DOACs [20], the 2.5-mg bid dose of rivaroxaban and aspirin is likely to have lower efficacy in this population than the standard 20 mg daily dose. Therefore, for PAD patients with atrial fibrillation, full-dose anticoagulation instead of the combination of low-dose rivaroxaban and aspirin should be used. Initial randomized trials assessing DOAC efficacy have revealed an elevated risk of bleeding when aspirin is added to DOAC in atrial fibrillation [39], and therefore aspirin should not be routinely prescribed to patients with stable PAD and atrial fibrillation who are receiving full-dose anticoagulant.

### MALE

As outlined above, questions remain regarding the optimal antithrombotic therapy following major adverse limb events. A subanalysis of the COMPASS trial revealed that 2% of enrolled PAD patients experienced MALE. In these patients, recurrent events were common and there was wide variation in subsequent antithrombotic therapy choices. The choice of optimal antithrombotics following a MALE event depends on the nature of the arterial atherothromboembolic event, the type of surgical intervention performed, and the patient’s bleeding risk. No studies have adequately investigated antithrombotic choice after acute limb ischemia (as defined as limb-threatening ischemia with evidence of acute arterial obstruction by radiological criteria or a new pulse deficit leading to an intervention—i.e., surgery, thrombolysis, peripheral angioplasty, or amputation—within 30 days of symptoms onset). There are a few small trials that have investigated antithrombotic choice post-vascular intervention. The largest of which is the DUTCH BOA trial, which found an overall increased bleeding risk, without a decrease in MACE or MALE, when utilizing warfarin (INR 3–4.5) as compared to aspirin following surgical bypass. Interestingly, anticoagulation was significantly better than aspirin in the subgroup of patients receiving vein grafts [18•, 19]. For patients undergoing endovascular intervention, studies have suggested a benefit of dual antiplatelet therapy with clopidogrel and aspirin over aspirin monotherapy [40], and current guidelines suggest at least 1 month of DAPT after infrainguinal stenting [6]. The more recent ePAD study found no difference in MACE and a trend towards decrease restenosis and revascularization with the combination of edoxaban and aspirin as opposed to clopidogrel and aspirin in patients with endovascular intervention [41]. The VOYAGER-PAD trial is currently investigating the benefit of rivaroxaban 2.5 mg twice daily added to antiplatelet therapy in patients undergoing infrainguinal surgical or endovascular revascularization [33]. This large randomized, double blind, placebo-controlled trial will help to better inform decision-making for these patients.

### Urgent Surgery

The concentration of rivaroxaban below which it should be considered safe to perform surgery is currently unknown. An international consensus statement considers a drug level of less than 30 ng/mL acceptable to undergo cardiac surgery, one of the surgical procedures with highest risk of bleeding. Although pharmacokinetic analyses on the 2.5-mg bid dose are few [42], studies of a 5-mg twice daily dose in healthy subjects suggest that median peak concentrations are likely to be around 40 ng/mL, with a peak time of 3 h after the dose and half-life of 7 h [43]. Patients with reduced creatinine clearance may have somewhat higher peak concentrations and longer time to clearance. It therefore seems reasonable to withhold rivaroxaban 2.5 mg for 12 h pre-operatively if possible, by which time, most patients will have levels below 30 ng/mL. In case of requirement for urgent surgery and a concern about bleeding, use of andexanet alfa (or prothrombin complex concentrate if andexanet is not available) could be considered.

## Conclusions

Patients with PAD have high rates of morbidity and mortality. Dual pathway inhibition with rivaroxaban and aspirin is effective at reducing the risk of MACE and MALE [7, 31]. With widespread approval by governing bodies, it should be strongly considered in PAD patients without high bleeding risk. Implementing the COMPASS regimen in patients with PAD, along with other vascular risk reduction strategies, will have a substantial impact on individual patient risk and overall population health.

Despite recent advancements, PAD continues to carry significant rates of morbidity and mortality. Much of this is due to underutilization of medical therapy [29,31]. Widespread commitment to the medical treatment of PAD is urgently required. Both existing and emerging therapies must be implemented by the vascular medicine and surgery community, working together to prevent cardiac events, limb events, and cardiovascular death.
